# Propofol and AZD3043 Inhibit Adult Muscle and Neuronal Nicotinic Acetylcholine Receptors Expressed in *Xenopus* Oocytes

**DOI:** 10.3390/ph9010008

**Published:** 2016-02-06

**Authors:** Malin Jonsson Fagerlund, Johannes Krupp, Michael A. Dabrowski

**Affiliations:** 1 Department of Anesthesiology, Surgical Services and Intensive Care Medicine, Karolinska University Hospital, SE-171 76 Stockholm, Sweden; 2 Department of Physiology and Pharmacology, Section for Anesthesiology and Intensive Care Medicine, Karolinska Institutet, SE-171 76 Stockholm, Sweden; 3 Department of Neuroscience, AstraZeneca Research and Development, CNS&Pain Innovative Medicines, AstraZeneca R&D, SE-151 85, Södertälje, Sweden

**Keywords:** anesthesia, intravenous anesthesia, propofol, AZD3043, nicotinic achetylcholine receptors

## Abstract

Propofol is a widely used general anaesthetic with muscle relaxant properties. Similarly as propofol, the new general anaesthetic AZD3043 targets the GABA_A_ receptor for its anaesthetic effects, but the interaction with nicotinic acetylcholine receptors (nAChRs) has not been investigated. Notably, there is a gap of knowledge about the interaction between propofol and the nAChRs found in the adult neuromuscular junction. The objective was to evaluate whether propofol or AZD3043 interact with the α1β1δε, α3β2, or α7 nAChR subtypes that can be found in the neuromuscular junction and if there are any differences in affinity for those subtypes between propofol and AZD3043. Human nAChR subtypes α1β1δε, α3β2, and α7 were expressed into *Xenopus* oocytes and studied with an automated voltage-clamp. Propofol and AZD3043 inhibited ACh-induced currents in all of the nAChRs studied with inhibitory concentrations higher than those needed for general anaesthesia. AZD3043 was a more potent inhibitor at the adult muscle nAChR subtype compared to propofol. Propofol and AZD3043 inhibit nAChR subtypes that can be found in the adult NMJ in concentrations higher than needed for general anaesthesia. This finding needs to be evaluated in an *in vitro* nerve-muscle preparation and suggests one possible explanation for the muscle relaxant effect of propofol seen during higher doses.

## 1. Introduction

Propofol (2,6-diisopropylphenol) is a widely used intravenous anesthetic for both general anesthesia and conscious sedation. The major determinant of the anesthetic effects of propofol is a positive allosteric modulation of GABA_A_ receptors in the CNS [1]. The GABA_A_ receptor belongs to the cys-loop family of the ligand-gated ion-channels composed by five transmembrane protein subunits assembled around a central pore. The central pore allows conduction of cat- or anions dependent on the receptor type. In the brain, most GABA_A_ receptor subtypes are composed of α, β, and γ or δ subunits, with the stoichiometry 2:2:1 (α:β:γ/δ) [2]. Many of the general anesthetics targeting GABA_A_ receptors have also affinity to other receptors of the cys-loop family such as the nicotinic acetylcholine receptors (nAChRs), glycine and 5-HT_3_-receptors [3]. More specifically, propofol inhibit fetal muscle (α1β1δγ) and the neuronal α4β2 nAChR subtype in concentrations higher than required for sedation and anesthesia [4,5]. To date, 17 nAChR subunits have been identified in mammals; the muscle α1, β1, δ, γ, and ε subunits, and the neuronal α2-10 and β2-4 subunits [6]. Intact transmission in the neuromuscular junction (NMJ) is dependent on both pre- and postsynaptic nAChRs; the presynaptic receptor being the α3β2 and the postsynaptic receptor present at the muscle membrane in adults is the α1β1δε, that in fetus is the α1β1δγ subtype or during denervation/inflammation the α7 nAChR subtype [7,8].

Propofol is well known to cause muscle relaxation in higher doses, which is a property that can be used to intubate the trachea without neuromuscular blocking agents [9]. It is established that propofol is the general anesthetic agent that provides the highest degree of muscle relaxation, however, the mechanism behind this is not fully characterized [10,11,12]. Since propofol inhibits the fetal and certain neuronal nAChRs, at least part of the muscle relaxant effect of propofol could theoretically be mediated by inhibition of nAChRs in the NMJ. Notably, it has not been verified if propofol indeed engages nAChRs present in the adult NMJ. Flood and co-workers have previously demonstrated that propofol has a low sensitivity for the α7 nAChR subtype but it has not been investigated whether propofol interacts with the adult α1β1δε subtype or the presynaptic α3β2 nAChR subtype. AZD3043 is a new rapid acting intravenous anesthetic agent currently in clinical trials and with similar effects as propofol on the GABA_A_ receptor, but the affinity for other receptor types has not been investigated in detail [13,14]. Thus, it has not been investigated whether AZD3043 inhibits nAChRs in the NMJ or how much muscle relaxation ADZ3043-based anesthesia provides.

The aim of this study was, therefore, to evaluate whether propofol or AZD3043 interact with the α1β1δε, α3β2, or α7 nAChR subtypes that can be found in the adult neuromuscular junction and if there are any differences in affinity for those subtypes between propofol and AZD3043.

## 2. Results

### 2.1. Basic Nicotinic Acetylcholine Receptor Receptor Pharmacology

All oocytes injected with the adult muscle or neuronal nAChR subtypes produced a concentration-dependent inward current in response to acetylcholine when voltage-clamped. All the expressed nAChR subtypes displayed typical currents upon activation with acetylcholine; very little desensitization at the α1β1δε nAChR subtype, the α3β2 nAChR subtype displayed a two-component pattern with a fast activation and desensitization, and thereafter a second, slower component. Rinse yields a partial release from desensitization. The α7 nAChR subtype displayed a fast, low current density. The acetylcholine EC_50_ value for the human α1β1δε nAChR subtype was 8.5 (7.2–10.0) μM as previously reported [15]. The EC_50_ value for the α3β2 subtype was 67.2 (14.4–312.5) μM and 49.4 (39.0–62.5) μM for the α7 nAChR subtype, which is similar to previously reported studies [16,17]. Uninjected oocytes did not respond to acetylcholine (data not shown).

#### 2.1.1. Effect of Propofol and AZD3043 on the Adult Human α1β1δε Nicotinic Acetylcholine Receptor Subtype

**Figure 1:**
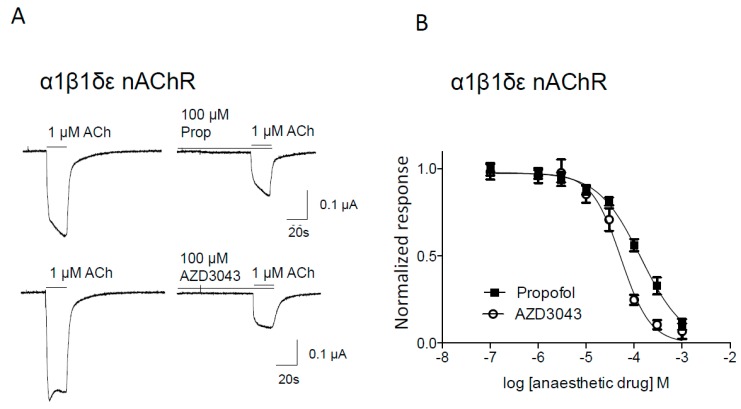
Propofol and AZD3043 concentration-dependently inhibit 1 µM acetylcholine-induced current responses in *Xenopus* oocytes expressing the human muscle (α1β1δε) nAChR. Representative traces from one oocyte inhibited by propofol (Prop) respectively AZD3043 (**A**). Concentration-dependent inhibition of currents induced by 1 µM ACh by propofol and AZD3043 (**B**). Current responses in each oocyte were normalized to the 1 µM acetylcholine control responses in each oocyte as described in *Materials and Methods*. Each symbol represents mean ± S.E.M. When no error bars are seen, they are smaller than the symbols. Ach = acetylcholine, Prop = propofol.

Application of 0.1–1000 μM propofol or AZD3043 did not elicit any current in the α1β1δε nAChR subtype (Figure 1A). However, both propofol and AZD3043 inhibited 1 μM acetylcholine-induced currents in a concentration-dependent and reversible manner (Figure 1).

AZD3043 had a lower IC_50_ compared to propofol and was, thus, a more potent inhibitor of the adult muscle nAChR subtype (Table 1).

**Table 1:** Pharmacological properties of propofol and AZD3043 on human nAChRs expressed in *Xenopus* oocytes.

nAChR Subtype	Drug	IC_50_ (95% C.I.) (μM)	nH (± SEM)	n/N
Muscle α1β1δε	Propofol	139 (112–172) **	0.97 ± 0.09	8/2
	AZD3043	53 (41–69)	1.41 ± 0.21	13/2
α7	Propofol	269 (200–362)	2.80 ± 1.49	8/2
	AZD3043	176 (59–527)	0.63 ± 0.23	5/2
Presynaptic α3β2	Propofol	118 (80–175)	0.95 ± 0.16	4/1
	AZD3043	38 (10–146)	0.84 ± 0.38	5/2

n/N = numbers of oocytes/number of batches. ** *p* < 0.01.

#### 2.1.2. Effect of Propofol and AZD3043 on the Human Neuronal α3β2 and α7 Nicotinic Acetylcholine Receptor Subtypes

**Figure 2:**
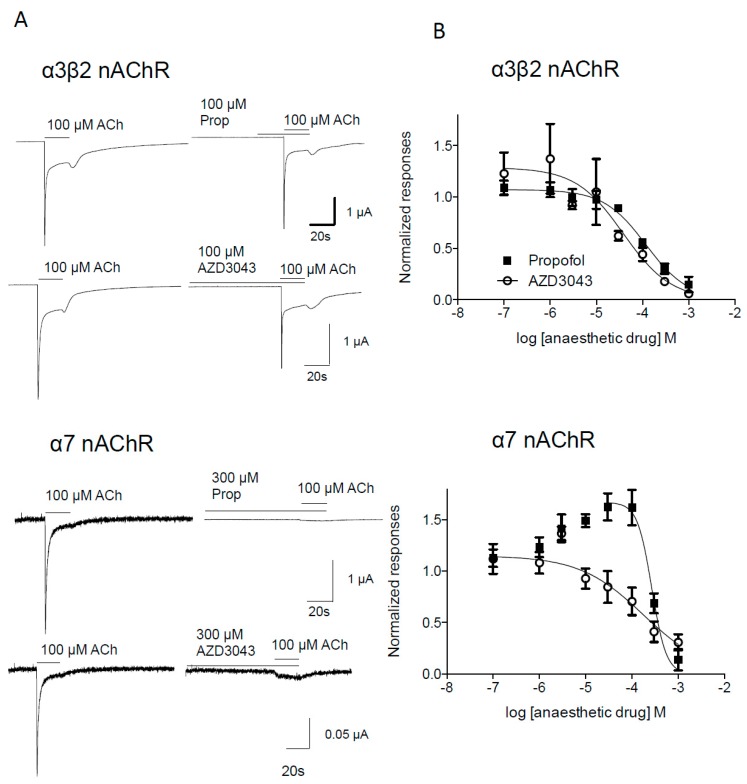
Propofol and AZD3043 concentration-dependently inhibit 100 µM acetylcholine-induced current responses in *Xenopus* oocytes expressing the human neuronal α3β2 and α7 nAChR. Representative traces from one oocyte inhibited by propofol (Prop) or AZD3043 (**A**). Please note that the concentration-response curve for the human neuronal α7 nAChR subtype is based on area under the curve calculation and not peak (see Section 4.6.). Concentration-dependent inhibition of currents induced by 1 µM acetylcholine by propofol and AZD3043 at the human neuronal α3β2 and α7 nAChR subtypes (**B**). Current responses in each oocyte were normalized to the 100 µM acetylcholine control responses in each oocyte as described in *Experimental Section*. Each symbol represents mean ± S.E.M. When no error bars are seen, they are smaller than the symbols. Ach = acetylcholine, Prop = propofol.

Neither propofol nor AZD3043 applied in the absence of acetylcholine elicited any currents at the α3β2 and α7 nAChR subtypes in concentrations from 0.1–1000 μM (Figure 2A). Similarly to the inhibition of α1β1δε, both propofol and AZD3043 inhibited 100μM acetylcholine-induced currents concentration-dependently and reversibly in the α3β2 and α7 nAChR subtypes (Figure 2). There were no significant differences between the IC_50_-values for propofol and AZD3043 at the human α3β2 and α7 nAChR neuronal subtypes, respectively (Table 1). Notably, in the lower concentration range of propofol (1–100 μM) a positive modulation of the α7 nAChR subtype was seen (Figure 2B). This was not noted for AZD3043 (Figure 2B).

## 3. Discussion

Here we demonstrate that propofol and AZD3043 inhibits human adult muscle and neuronal α3β2 and α7 nAChR subtypes expressed in *Xenopus* oocytes. Moreover, AZD3043 was a more potent inhibitor at the adult muscle nAChR subtype compared to propofol. For the neuronal nAChR subtypes there were no differences in inhibition between both anesthetics.

It has been known for more than 20 years that propofol inhibits the fetal α1β1δγ nAChR subtype expressed in *Xenopus* oocytes [5], and recently the binding site for propofol in the fetal subtype has been characterized [18]. Thus, it is well-established that propofol inhibits and binds to the fetal nAChR. However, the effect of propofol on the adult α1β1δε nAChR subtype has not been investigated previously. Based on the results of this study, propofol is a 2–3 times more potent inhibitor on the fetal subtype compared to the adult, with an IC_50_-value of 46 μM for the fetal type [5] *vs.* 139 μM for the adult type.

To our knowledge, the effect of propofol on the α3β2 nAChR subtype has not been studied before. Notably, the effect of propofol on this subtype is relevant since the α3β2 nAChR subtype is a presynaptic autoreceptor in the NMJ, and inhibition of this receptor is suggested to cause the train-of-four fade during a non-depolarizing neuromuscular blockade [7,17]. Propofol is a less potent inhibitor at this presynaptic α3β2 nAChR subtype and also at the α7 nAChR subtype, compared to the muscle α1β1δε nAChR subtype. Our demonstration of an apparent full inhibition by a high concentration of propofol of the α7 subtype is in contrast to Flood and co-workers who only demonstrated a 5% inhibition [19]. A possible explanation for this difference is that Flood and co-workers studied the effect of propofol (400 μM) only at 1 mM acetylcholine, a concentration that is well above the acetylcholine EC_50_ for this receptor [5]. Since it is well known that the α7 receptor rapidly desensitizes after exposure to high concentrations of agonist [19], it is maybe not surprising that the modulation by propofol would be different between our study and the study by Flood and co-workers. Indeed, we find a dual modulatory effect of the α7 nAChR subtype by propofol: there is a positive modulation of the receptor with concentrations up to 100 μM propofol, and a inhibition of the α7 subtype at propofol concentrations of 300 μM and higher.

The effect of AZD3043 on nAChR has not been investigated before [14], but is more potent in inhibition of the adult muscle nAChR compared to propofol. AZD3043 shares the same properties as propofol in terms of inhibition of the α3β2 nAChR subtype, but lacks the positive modulation of the α7 subtype seen with propofol although the IC_50_ values were similar. Whether this difference in effect of the α7 subtype between AZD3043 and propofol in the lower concentration range translates to a clinical finding needs to be explored.

The affinity to several receptors other than those that are the primary targets for general anesthesia may give rise to side effects of general anesthetics, but may also have potentially-advantageous properties [3]. One of these is the muscle relaxant effect of propofol when given in higher doses [9]. It is generally accepted that propofol is the general anesthetic with the highest muscle relaxant effect on laryngeal and pharyngeal muscles, however the exact mechanism behind the muscle relaxation properties of propofol is not fully understood and conflicting data exists [20,21,22]. In order to evaluate the relevance of the propofol- and AZD3043-induced inhibition of the nAChR subtypes studied here, the IC_50_ values needs to be correlated with clinically relevant concentrations for both drugs. If propofol is used as a single agent, the EC_50_ for propofol in humans to prevent movements to surgical incision is 15.2 μg/mL [23] and steady state concentrations during surgery ranges from 2.5–6 μg/mL (corresponding to a free aqueous concentration of 0.25–0.6 μM). Thus, the inhibition of the nAChR by propofol is above the clinical concentrations ranges, although it is difficult to compare plasma concentrations in the clinical setting with *in vitro* values. Therefore, it is more relevant to compare the effect of propofol and AZD3043 at their main target in a similar *in vitro* system, *i.e*, the effect on the GABA_A_ receptor. The EC_50_ values for propofol and AZD3043 at relevant GABA_A_ receptors are in the range between 14–25 μM and 30–34 μM, respectively [13,24,25,26]. There are no plasma concentrations in humans available for AZD3043 published to date. The IC_50_ values presented here for propofol and AZD3043 at the nAChR subtypes studied are higher compared to the concentrations needed for a positive modulation of the GABA_A_ receptor, but are comparable to the exposure after a high bolus dose of propofol given during induction.

The *in vitro* data presented here also needs to be correlated with effects measured in the intact NMJ of animal and in humans. However, if the proposed muscle relaxant effects during induction can be confirmed in such systems a potential use of AZD3043 could be as an intravenous anesthetic with muscle relaxant properties in addition to the predicted anesthetic effect. Again, this needs to be confirmed in animal and human models and, therefore, more studies are warranted both on AZD3043 and propofol in this aspect.

## 4. Materials and Methods

### 4.1. Clones

The human nAChR subunits α1, α3, α7, β1, β2, δ, and ε were cloned from a human cDNA library, and the cDNAs were subsequently subcloned into different expression vectors, pKGem (AstraZeneca, Wilmington, DE, USA) (α1, α3, β1, β2, δ, and ε) and pBluescript II SK (−) (Stratagene, La Jolla, CA, USA) (α7) [15,16,17]. Messenger RNA was transcribed *in vitro* using the mMessage mMachine^®^ T7 kit (Ambion, Austin, TX, USA) and analyzed using a bioanalyzer (Agilent Technologies, Palo Alto, CA, USA).

### 4.2. Xenopus Oocyte Injection

Ethical approval for this study (S34/07) was provided by the South Local Animal Ethics Committee of Stockholm, Sweden, (Chairperson Barbro Ahlbeck) on 27 April 2007. Preparation and injection of oocytes and the electrophysiological recordings were done as previously described [15,16,17]. Briefly, *Xenopus laevis* oocytes were isolated by partial ovariectomy from frogs anesthetized with 0.2% Tricaine. The ovaries were mechanically dissected to smaller lumps and digested in OR-2 buffer (in mM, NaCl 82.5, KCl 2, MgCl_2_ 1, HEPES 5, pH adjusted to 7.5 with NaOH) containing 1.5 mg/mL collagenase (Type 1A, Sigma, St. Louis, MO, USA), for 90 min in order to remove the follicular epithelia from the oocytes. After 1–24 hours the oocytes were injected with 0.2–18 ng mRNA in a total volume of 30–40 nL/oocyte. The α1β1εδ or α3β2 subtypes were injected at a 1:1 ratio. The oocytes were maintained in Leibovitz L-15 medium (Sigma, St. Louis, MO, USA) diluted 1:1 with Millipore-filtered ddH_2_O (Billerica, MA, USA) and 80 μg/mL gentamycin, 100 units/ml penicillin, and 100 μg/mL streptomycin added. Oocytes were incubated at 18–19 °C for 2–7 days after injection before being studied. 

### 4.3. Electrophysiological Recordings

All recordings were performed at room temperature (20–22 °C). During recording, the oocytes were continuously perfused with ND-96 (in mM), NaCl 96.0, KCl 2.0, CaCl_2_ 1.8, MgCl_2_ 1.0, HEPES 5.0, pH 7.4 adjusted with NaOH. Oocyte recordings were performed using an automated two-electrode voltage clamp system (OpusXpress 6000A, Molecular Devices, Sunnyvale, CA, USA) as previously described [16,17]. Electrodes were made from 1.5 mm borosilicate tubes (World Precision Instruments Inc, Sarasota, FL, USA) and filled with 3 M KCl (0.5–2.5 MΩ resistance). The oocytes were voltage clamped at −60 mV.

### 4.4. Protocol

Oocytes were continuously perfused with ND-96 at a rate of 2 mL/min in a 150 µL chamber. Drugs were delivered from a 96 well plate using disposable tips and administered at a rate of 2 mL/min for the first 2 s, and thereafter at 1 mL/min. Concentration–response curves for acetylcholine were constructed for each subunit in order to determine the EC_50_. To determine whether propofol and AZD3043 activate and, furthermore, inhibit acetylcholine-induced currents, these drugs were applied for 55 s prior to a 20 s co-application of both anaesthetic and acetylcholine. Between each drug application, there was a 6 min washout period, to allow clearance of the drugs and to avoid desensitization of the channels. Before and after each concentration–response experiment, three acetylcholine-control responses were recorded in order to exclude desensitization. Experiments were rejected if the post-control response was less than 80% of the pre-control response. In order to adjust for the level of channel expression, the responses in acetylcholine concentration–response experiments were normalized to the peak response in each individual oocyte. For inhibition experiments, responses in each oocyte were normalized to the mean of the second and third acetylcholine pre-controls.

### 4.5. Drugs

Acetylcholine and propofol were purchased from Sigma (St. Louis, MO, USA). AZD3043 was synthesized by AstraZeneca (AstraZeneca, Södertälje, Sweden) [13]. Chemicals used in buffers were purchased from Sigma (St. Louis, MO, USA) or Merck (Nottingham, UK) unless otherwise stated. Stock solution of 1 mM acetylcholine was prepared in ND-96 buffer and frozen. Stock solutions of 100 mM propofol and 100 mM AZD3043 were made fresh in 100% DMSO. In order to avoid own effects by DMSO [27], the maximal concentration of DMSO in ND-96 at 1 mM of drug was <0.1%, where no inhibitory effects has been seen on the nAChRs investigated in this study. All drugs were then diluted in ND-96 immediately before use.

### 4.6. Statistical Analysis

Off-line analyses were made using Clampfit 9.2 (Molecular Devices, Sunnyvale, CA, USA). Changes in current were studied as peak for the α1β1δε and α3β2 nAChRs and as net charge (area under the curve) for the α7 nAChR due to rapid desensitization [16,17,28]. The baseline current immediately before drug application was subtracted from the response, and the analysis region was 20 s, *i.e.*, the time of agonist application. Concentration–response relationships for acetylcholine were fitted by non-linear regression to the four-parameter logistic equation (Prism 6.0, GraphPad, San Diego, CA, USA):

$$Y=\text{Bottom}+\frac{\text{Top}-\text{Bottom}}{\left[1+{\left(\frac{x}{{\text{EC}}_{50}}\right)}^{\text{nHill}}\right]}$$

where Y is the normalized response, x is the logarithm of concentration, and EC_50_ is the logarithm of the concentration of agonist eliciting half-maximal response. When propofol and AZD3043-induced inhibition was studied, the same equation was used and EC_50_ was replaced by IC_50_, which is the concentration of antagonist eliciting half maximal inhibition. Data are presented as mean ± S.E.M or 95% confidence interval (95% CI) as appropriate. The concentration–response curves are global fits of all oocytes, whereas the IC_50_ values were analyzed statistically on the basis of individual oocyte fits that were analyzed as sets. IC_50_ values for the effect of propofol and AZD3043 in individual oocytes were compared using independent t-tests for each nAChR subtype. GraphPad Prism (Prism 6.0, GraphPad, San Diego, CA, USA) was used for statistics and graphs. A *P*-value of <0.05 was considered significant.

## 5. Conclusions

This study demonstrates that propofol and AZD3043 inhibit nAChR subtypes that can be found in the adult NMJ in concentrations higher than needed for general anaesthesia. This finding needs to be evaluated in an *in vitro* nerve-muscle preparation and suggests one possible explanation for the muscle relaxant effect of propofol seen during higher doses.

## Abbreviations

The following abbreviations are used in this manuscript:

nAChR: Nicotinic acetylcholine receptor
NMJ: Neuromuscular junction
